# Implications of dual practice for universal health coverage

**DOI:** 10.2471/BLT.14.151894

**Published:** 2015-11-19

**Authors:** Barbara McPake, Giuliano Russo, David Hipgrave, Krishna Hort, James Campbell

**Affiliations:** a Nossal Institute for Global Health, University of Melbourne, Melbourne, Australia.; b Instituto de Higiene e Medicina Tropical, Nova University of Lisbon, Rua da Junqueira 100, Lisbon, 1349-008, Portugal.; c UNICEF, New York, United States of America.; d World Health Organization, Geneva, Switzerland.

## Abstract

Making progress towards universal health coverage (UHC) requires that health workers are adequate in numbers, prepared for their jobs and motivated to perform. In establishing the best ways to develop the health workforce, relatively little attention has been paid to the trends and implications of dual practice – concurrent employment in public and private sectors. We review recent research on dual practice for its potential to guide staffing policies in relation to UHC. Many studies describe the characteristics and correlates of dual practice and speculate about impacts, but there is very little evidence that is directly relevant to policy-makers. No studies have evaluated the impact of policies on the characteristics of dual practice or implications for UHC. We address this lack and call for case studies of policy interventions on dual practice in different contexts. Such research requires investment in better data collection and greater determination on the part of researchers, research funding bodies and national research councils to overcome the difficulties of researching sensitive topics of health systems functions.

## Résumé

Pour parvenir à la couverture sanitaire universelle, il est nécessaire que les agents sanitaires soient en nombre suffisant, préparés à exercer leur métier et motivés. Au moment de déterminer les meilleurs moyens de renforcer le personnel de santé, peu d'attention a été accordée aux tendances et aux conséquences de la double pratique (cumul d'emplois dans les secteurs public et privé). Nous avons examiné une étude récente sur la double pratique en raison de sa capacité à orienter les politiques de dotation en personnel par rapport à la couverture sanitaire universelle. De nombreuses études décrivent les caractéristiques et les corrélats de la double pratique et suggèrent des hypothèses quant à son impact, mais très peu de données présentent un intérêt direct pour les dirigeants. Aucune étude n'a évalué l'impact des politiques sur les caractéristiques de la double pratique ou les conséquences sur la couverture sanitaire universelle. Nous soulignons ce manque et recommandons la réalisation d'études de cas portant sur les actions politiques relatives à la double pratique dans différents contextes. Ce travail de recherche requiert une meilleure collecte de données et une plus grande détermination de la part des chercheurs, des organismes de financement de la recherche et des conseils nationaux de recherche pour surmonter les difficultés liées aux recherches sur des sujets délicats concernant les fonctions des systèmes de santé.

## Resumen

Progresar hacia una cobertura universal de salud (UHC, por sus siglas en inglés) requiere un número adecuado de personal sanitario que esté preparado para su trabajo y motivado para llevarlo a cabo. Al establecer las mejores formas para desarrollar el personal sanitario, se prestó relativamente poca atención a las tendencias e implicaciones de la doble práctica (empleo concurrente en los sectores públicos y privados). Se revisó una reciente investigación sobre la doble práctica por su potencial para guiar las políticas de personal en relación con la UHC. Muchos estudios describen las características y correlaciones de la doble práctica y especulan sobre sus efectos negativos, pero existen muy pocas pruebas de que sean directamente relevantes para los responsables políticos. Ningún estudio ha evaluado el impacto de las políticas en las características de la doble práctica o las implicaciones para la UHC. Se señala esta carencia y se reclaman estudios de casos de las intervenciones políticas sobre la doble práctica en diferentes contextos. Tal investigación requiere una inversión en una mejor recopilación de datos y una mayor determinación por parte de los investigadores, las entidades que financian la investigación y los consejos nacionales de investigación con el objetivo de superar las dificultades de investigar temas delicados del funcionamiento de los sistemas sanitarios.

## ملخص

إن تحقيق التقدم في اتجاه التغطية الصحية الشاملة (UHC) يتطلب أن يكون العاملون بمجال الخدمات الصحية بأعداد كافية، ومستعدين لتأدية أعمالهم، ومتحفزين للقيام بأداء جيد. ****ومن أجل تحديد أفضل السبل لتطوير القوى العاملة في مجال الصحة، تم إيلاء اهتمام قليل نسبيًا بالنزعات والتأثيرات المترتبة على "الممارسة المزدوجة"، والتي تشير إلى تولي العمل في القطاعين العام والخاص في نفس الوقت. ****إننا نراجع البحث الجاري مؤخرًا عن الممارسة المزدوجة بسبب احتمالية توجيهه لسياسات التوظيف فيما يتعلق بالتغطية الصحية الشاملة. ****وتوضح العديد من الدراسات الخصائص والعوامل المرتبطة بالممارسة المزدوجة وتتكهن حول التأثيرات، ولكن توجد أدلة قليلة جدًا مرتبطة بشكل مباشر بصناع القرار. ****لم تقم أية دراسات بتقييم تأثير السياسات على خصائص الممارسة المزدوجة أو التأثيرات للتغطية الصحية الشاملة. ****إننا نتناول عامل النقص هذا وندعو لإجراء دراسات حالة لتدخلات السياسة في الممارسة المزدوجة في ظروف مختلفة. ****يتطلب هذا البحث الاستثمار في وسائل أفضل لجمع البيانات وعزيمة أكبر من جانب الباحثين، وهيئات تمويل البحوث، ومجالس للبحوث الوطنية للتغلب على صعوبات إجراء بحوث عن مواضيع حساسة لوظائف الأنظمة الصحية.

## 摘要

若要在全民医疗保障 (UHC) 方面取得进展，则需医疗工作者人数充足、对其工作准备充分并具有执行积极性。 为了确立培养医疗工作者的最佳途径，人们较少关注双重实践（公立和私营单位同时展开）的发展趋势和所受影响。 针对全民医疗保障 (UHC) 人员招募指导政策对双重实践的潜在影响，我们查看了近期研究。很多研究在影响方面对双重实践与实践推断的特性和关联进行了描述，但是很少有证据表明双重实践受到的影响与政策制定有直接联系。 尚无研究围绕招募政策对双重实践特性的影响或招募政策对全民医疗保障 (UHC) 的影响进行评估。 我们提出了这一不足，并且提倡从不同方面对双重实践进行政策干预案例研究。 需要对此类研究投入资金，以便实现更好的数据采集，并让研究工作者、研究资助机构和国家研究委员会更有决心克服困难，从而对涉及医疗系统功能服务的敏感性课题进行研究。

## Резюме

Развитие всеобщего охвата услугами систем здравоохранения (ВОУСЗ) требует достаточного количества хорошо подготовленных и мотивированных медицинских работников. В методиках повышения качества развития работников здравоохранения относительно мало внимания уделяется тенденциям и значению двойной практики, а именно тем случаям, когда врач одновременно работает в государственном и частном секторе здравоохранения. Было проанализировано недавнее исследование двойной практики, посвященное ее потенциальной возможности направлять кадровую политику на то, что касается ВОУСЗ. Во многих исследованиях описываются особенности и корреляты двойной практики и рассматривается ее воздействие, но фактических данных, непосредственно относящихся к лицам, принимающим решения, крайне мало. Исследования, в которых изучалось бы воздействие мер политики на особенности двойной практики или на значение для ВОУСЗ, отсутствуют. Данная статья — это попытка заполнить этот пробел путем рассмотрения тематических исследований мер политики, предпринятых в отношении двойной практики в различных условиях. Такие исследования требуют принятия мер по усовершенствованию сбора данных, а также большей решимости со стороны исследователей, органов, финансирующих исследования, и национальных исследовательских советов, чтобы преодолеть трудности в изучении важных вопросов относительно функционирования систем здравоохранения.

## Introduction

Over the last five years, universal health coverage (UHC) has become an agreed goal of global health policy and planning initiatives.^1^^,^^2^ Target 3.8 of the Sustainable Development Goals (SDGs) is to achieve UHC, including financial risk protection, access to quality essential health-care services and access to safe, effective, quality and affordable essential medicines and vaccines for all.^3^ However, scholars and health policy-makers have noted that attaining this goal will require a sufficient number of prepared and motivated health workers.^4^ Others have drawn attention to the importance of harnessing labour market forces to achieve UHC.^5^^,^^6^

The World Health Organization (WHO) is developing a global strategy on human resources for health.^7^ A consultation has concluded that progress towards UHC will require integrated, people-centred health services, a motivated health workforce and adequate financing from domestic and other sources. While the importance of human resources in UHC and the SDG agenda has been recognized, the extent and impact of health workers’ dual practice – that is, concurrent clinical practice in public and private sectors^8^ – has not received much attention.^9^ However, given the pervasiveness of dual practice and the growing prominence of the private sector in the provision of health services worldwide,^10^ its dynamics and impact on the attainment of UHC should not be ignored. There are many unanswered questions about the impact and consequence of dual practice. Especially in low- and middle-income countries, the achievement of UHC may be hampered by unregulated dual practice.^5^

Both the public and private sectors are needed to attain UHC.^11^ Failure to understand why, how and to what extent health workers engage in dual practice may compromise attempts to regulate it and undermine progress. Here we present dual practice examples, focusing on UHC-associated policy relevance of the available evidence, especially in low- and middle-income countries. We consider regulatory options in a range of contexts and consider future research needs.

## Dual practice

Dual practice is common among health professionals worldwide.^12^^–^^14^ In the United Kingdom of Great Britain and Northern Ireland, over 60% of public hospital doctors conduct private practice alongside their National Health Service work;^15^ in Spain, 20% of public sector doctors have a second job^16^ and about 25% of public hospital doctors in Norway reported holding a private sector job.^17^ Dual practice is reported to be very common among certain categories of specialized nurses.^18^ In Portugal, public sector physicians and nurses can choose to be employed on exclusive contracts with better pay or on contracts with reduced pay that permit private sector work.^12^

Dual practice is widespread in Latin America^19^ as well as in Asia, where it may be rising with increasing levels of both health expenditure and private sector participation in Asian health systems.^20^ In Bangladesh, most doctors – particularly specialists – earn more than half of their income in private practice.^21^ Over 80% of public sector physicians have been found to engage in private activities in Egypt, Indonesia, Kenya and Mexico.^8^ In a sample of South African public hospitals, about 40% of nurses reported working for the private sector.^22^ In six African, Asian and Central American countries, nurses and midwives were less likely to engage in dual practice than physicians.^23^

There are several forms of dual practice. Health professionals can work in a public service provision role and another role: (i) outside: in a completely separate private environment; (ii) beside: in a private ward or clinic physically associated with a public facility but run as a separate business; (iii) within: where private services are offered inside a public facility but outside public service operating hours or space; or (iv) integrated: where additional fees are charged for services offered alongside standard public ones, often informally, on the understanding of a faster – or higher quality – service.^24^ Academics and policy-makers typically restrict the term dual practice to category (i), but it is clear that categories (ii) to (iv) offer a range of options for health professionals and policy-makers to combine public and private practice, to supplement public sector salaries and retain personnel. Decisions health professionals make on their practice will be influenced by the range of opportunities across the categories and a line drawn between any two categories in the four-category spectrum may be an artificial distinction.

### Labour market decision-making

Standard labour market theory anticipates a supply curve whereby in most cases, suppliers of labour increase the hours they are willing to work as the wage rate increases. Although this supply curve does apply to health professionals,^6^ many of these professionals may be able to determine the prices of their services. As providers with a degree of monopoly power, they may not be constrained by a supply curve and may choose to provide more services at a lower price to gain market share while others offer fewer services at a higher price, thus reversing the expectations (and the causal direction) of the supply curve. This may explain the observed lack of relationship between the wage rate and number of hours worked by dual practitioners in the private sector from a recent study.^25^ However, this same study showed that the decision to practice in both sectors was predicted by potential levels of earning in the private sector and specific physicians’ personal characteristics.^25^ This observation suggests that while economic factors are important, increased public sector pay may not suffice to reduce dual practice.

### Impact of dual practice

There is widespread concern that dual practice reduces the accessibility and/or quality of care available to users of the public system.^26^ While in principle, absenteeism, over-servicing or deliberate creation of a quality gap between the public and private service might occur, it can equally be claimed that opportunities for public-sector health professionals to practice privately keep them in the national labour market and/or in the public sector. Without such opportunities, they might migrate overseas, move into full-time private practice or into other professions.^27^

The implications of dual practice are likely to be context-specific, dependent on the regulatory environment and opportunities for dual practice, demand for public and private services, various workplace environments and economic, social and cultural characteristics of the population and its health workforce.^20^ Dual practice can be beneficial in health systems with a capacity to enforce regulations that protect free-of-charge public services. However, in the absence of such regulation, public services might be adversely affected. A few cases suggest that dual practice has improved overall access to health care, while other research indicates that the opportunities for dual practice contribute to an urban bias in doctors’ distribution.^28^ However, studies have been limited by the absence of a clear counterfactual: how would the health system function in the absence of dual practice? Clearly there is more than one factor for governments to consider when assessing the implications of dual practice.

### Intervention and regulation

Analysis of opportunities for policy and regulation rests on the argument that some kind of intervention is needed to ensure positive outcomes. Unfortunately, no research has evaluated the impact of alternative interventions. However, it is possible to map current regulatory strategies against a logical set of options: (i) take no action; (ii) ban or limit dual practice and (iii) allow dual practice but regulate behaviour in public and private spheres.^14^^,^^20^ Most governments favour either taking no action or limiting dual practice, but engaging civil society in the regulation of health professionals’ behaviour may be more promising. The right to affordable health care is increasingly expected in societies when these are governed by locally-accountable authorities. Potential policy options to regulate dual practice range from command and control, to meta-regulation, self-regulation, market-mechanisms and voluntarism.^20^^,^^29^

A typology of dual practice may help to clarify policy options.^14^^,^^30^ Table 1 lists dual practice situations, consequences for UHC goals and respective regulatory options, based on a framework linking forms and prevalence of dual practice to local market and health governance conditions, as well as to characteristics of health workers.^24^

**Table 1:** Dual practice typology: examples of local conditions, consequences for UHC goals and policy options

Local conditions	Types of dual practice observed	Country example	Potential negative consequences for UHC goals	Type of regulatory options
– Limited ability and willingness to pay for health services – Limited private sector development – Blurred boundaries between public and private, large informal private sector – Poor regulation and enforcing capacity	Pervasive and unregulated dual practice, present in all its forms – outside, beside, within, as well as integrated to public services^24^	Bangladesh,^21^ Guinea Bissau,^24^ Nepal, Peru^19^	– Reduced provision of free-of-charge services – Absenteeism and shirking by public sector health workers – Illegal charges in public facilities	– Introduce top-down regulation limiting health workers’ dual practice – Inform public patients about fees and charges, including free-of-charge services – Separate public and private services

– Rising incomes and ability to pay for health services – Improved governance, regulatory and implementation capacity – Incipient formal private sector clearly separated from the public sector	Dual practice to some extent regulated, and present outside and beside and at times within public services, but not in its integrated form	Cabo Verde, China,^31^ Mozambique,^24^^,^^25^ South Africa,^22^ Thailand^27^	– Poor quality public services – Diversion of public patients to private practices – Public sector personnel disproportionally distributed in facilities or locations in which dual practice is possible – Limited range of public health services	– Allow regulated dual practice outside and inside public facilities in specific places and times – Monitor the implementation of regulation – Offer exclusivity contracts – Encourage self-regulation by professional bodies

– High-income – Sophisticated health systems and regulatory capacity – Established private sector	Regulated dual practice, allowed outside, and in some instances, beside public services	Australia, Canada,^8^ Italy, Portugal,^12^ Spain,^16^ United Kingdom^15^	– Poor quality public services – Diversion of public patients to private practices – Public-sector health workers move to the private sector	– Market-based or financial interventions – Provide incentives for positive behaviour – Regulation by professional bodies – Provide incentives to the private sector when outsourcing services – Establish contracts with private providers

UHC: universal health coverage.

In those countries with limited ability to remunerate public sector staff, less developed health markets, weak regulatory capacity and porous public–private boundaries, unregulated private activities tend to spread to public facilities. This reduces the provision of free-of-charge services. In such cases policy options could include top-down government regulation of dual practice, separation of private services and informing patients of their rights to access care without being charged.

For those countries (e.g. Cabo Verde, China, South Africa and Thailand) with increasing demand for private services, increasing regulatory capacities and increased private sector provision, dual practice poses the risk of diverting patients and health professionals to the private sector.^22^^,^^24^^,^^25^^,^^27^^,^^31^ In these circumstances, measures that seem to have been effective in protecting UHC goals are: (i) allowing regulated private services outside or beside, but not within, public services; (ii) closely monitoring the implementation of such practices; (iii) offering health workers better terms through exclusivity contracts; and (iv) supporting professional bodies in regulating the practice of their members.

Lastly, in high-income countries with sophisticated health systems, established private sectors, strong and independent regulatory capacity and empowered patient advocacy groups, regulatory efforts should be aimed at helping the market enhance breadth and quality of available services, as well as at retaining health personnel in the public sector. Potential options include (i) regulating public–private partnerships; (ii) building positive incentives into contracts to outsource public services to the private sector; and (iii) giving professional councils a primary role in defining the boundaries of dual practice.^12^^,^^15^^,^^16^

From the perspective of UHC, the core objective must be ensuring population coverage of a comprehensive package of health services, while guaranteeing social protection. Such services can be enhanced and complemented – but not replaced – by private expenditure and private provision. Contextual factors that might shape the approach to ensuring this complementarity include the availability of health workers, their opportunities for mobility and migration, the socioeconomic characteristics of the population served, the stage of private sector development, the development of health insurance institutions, regulatory capacities and the existence of – or ability to create – institutions to manage dual practice beside and within public institutions. The complexity of these variables suggests that a subjective, simplified dichotomy (good/bad) and a global prescription about regulatory approaches are unlikely to be helpful. Case studies of regulation in context would improve our understanding of the policy options and the factors affecting their impacts on a case-by-case basis.

## Conclusion

Understanding the prevalence, forms and regulation of dual practice is crucial for the achievement of UHC. Recent studies have added to the evidence base on health professionals’ simultaneous provision of public and private services in high-income as well as low- and middle-income countries, but have also highlighted research gaps. Given the prevalence of dual practice, a better understanding of the factors that influence health workers’ labour market decisions is needed. It can be difficult to research and regulate in an area in which holding a second job, if not illegal, is viewed as an ethically dubious practice.

We have offered a typology of health workers’ dual practice from different contexts, highlighting implications for UHC goals and possible policy options. As noted in WHO’s draft *Global strategy on human resources for health: workforce 2030*,^32^ improved collection and use of health workforce data for planning and policy-making is needed. Such data, to be consolidated through the application of a national health workforce account, should include information on workforce distribution, flows, demand, supply and remuneration, in both public and private sectors. Investment in better data collection and analysis is a prerequisite to designing and monitoring appropriate regulation of dual practice. Without such evidence, only approximate efforts can be made towards attaining UHC. People who are unable to access health professionals in their private roles may not receive the health services they need.

The lack of a single study on the impact of any regulatory approach to dual practice is further evidence of the neglect of areas of health systems research where questions are difficult to address. To ensure progress towards UHC, researchers, funding bodies and national research councils will need to overcome the difficulties of researching this aspect of the health system.
